# Direct Production of 2-Butanol from Glucose by Recombinant *Klebsiella pneumoniae* Strains

**DOI:** 10.3390/ijms27062892

**Published:** 2026-03-23

**Authors:** Emanoel Gergov, Alexander Arsov, Kaloyan Petrov, Lidia Tsigoriyna, Penka Petrova

**Affiliations:** 1 Institute of Microbiology, Bulgarian Academy of Sciences, 1113 Sofia, Bulgaria; emanoelgergov@microbio.bas.bg (E.G.); al.arsov@microbio.bas.bg (A.A.); 2 Institute of Chemical Engineering, Bulgarian Academy of Sciences, 1113 Sofia, Bulgaria; lidia@iche.bas.bg

**Keywords:** 2-butanol, *Klebsiella pneumoniae*, *Lentilactobacillus diolivorans*, *Clostridium beijerinckii*, diol dehydratase, alcohol dehydrogenase

## Abstract

2-Butanol is a promising biofuel due to its favorable properties and lower microbial toxicity compared to other butanol isomers. However, microbial production remains challenging due to the absence of a native biochemical pathway for directly converting sugars into 2-butanol. To achieve this goal, glucose should be directed through the 2,3-butanediol (2,3-BD) pathway, involving α-acetolactate synthase, α-acetolactate decarboxylase, and butanediol dehydrogenase for the formation of meso-2,3-BD, followed by diol dehydratase-catalyzed conversion of meso-2,3-BD to butanone and alcohol dehydrogenase-mediated reduction in butanone to 2-butanol. In this study, we report the development of six new recombinant strains based on *Klebsiella pneumoniae* G31, in which the metabolic pathway for converting glucose to meso-2,3-BD was extended to 2-butanol. All engineered strains harbored the vitamin B_12_-dependent diol dehydratase complex (*pduCDEGH*) from *Lentilactobacillus diolivorans* DSM 14421 under its native promoter control. In addition, *pduQ* from the same strain, and *adh* from *Clostridium beijerinckii* DSM 51 encoding alcohol dehydrogenases were expressed under native, T7, or P*tac* promoters. The highest yield of 2-butanol from glucose was achieved by *K. pneumoniae* K6 carrying the *adh* gene under the control of the T7 promoter—437 mg/L. Using 2-butanone as a substrate, K6 again produced the highest titer of 2-butanol (3.9 g/L), followed by the recombinant K8 (with *adh* under the P*tac* promoter), and notably, by the native *K. pneumoniae* strains. Therefore, although *pduQ* encodes a key alcohol dehydrogenase in *L. diolivorans*, it has weaker properties than *adh* for the *K. pneumoniae* host in all promoter configurations. As the high expression levels of *adh* under T7 promoter control were driven by the native bacterial RNA polymerase, this promoter–host combination appears particularly suitable for developing other strains of industrial relevance.

## 1. Introduction

The depletion of fossil fuel resources and the ongoing global energy crisis have necessitated the search for sustainable, renewable biofuels [1,2,3,4]. Among higher alcohols, butanol is considered one of the most promising next-generation biofuels [5], due to its high energy density, low hygroscopicity, and compatibility with existing fuel infrastructure [6]. The chemical synthesis of butanol has traditionally relied on established industrial and laboratory methods. Conventional 2-butanol production involves the hydration of petroleum-derived 2-butene, achieved either via indirect sulfuric acid ester formation and hydrolysis or through direct acid-catalyzed hydration under high temperature and pressure [7]. At the laboratory scale, high selectivity is obtained through Grignard reactions between ethyl magnesium bromide and acetaldehyde, as well as through the reduction of butanone using metal hydrides or catalytic hydrogenation [8]. Although effective, these chemical routes typically require energy-intensive conditions and hazardous reagents, generating substantial chemical waste and a larger environmental footprint than bio-based alternatives [9,10]. In contrast, microbial synthesis from renewable substrates under mild conditions offers a more sustainable approach consistent with green chemistry principles [11]. Among the four butanol isomers, microbial production has largely focused on 1-butanol; however, industrial-scale production remains limited by complex regulation, byproduct formation, cellular toxicity, and costly downstream processing [12,13]. The physicochemical properties of 2-butanol are comparable to those of 1-butanol, while its significantly lower microbial toxicity represents a distinct advantage for biotechnological production systems [14,15].

Nevertheless, microbial production of 2-butanol is exceedingly rare in nature. Only a few lactic acid bacteria are known to produce it, and even among those, the ability is quite rare. Out of 42 lactic acid bacterial isolates from nine species, only *Levilactobacillus brevis* was found to convert meso-2,3-butanediol (meso-2,3-BD) into 2-butanol [16], followed by the discovery of this capacity in *Lentilactobacillus diolivorans* [17,18]. Russmayer et al. reported that, since *L. diolivorans* cannot synthesize meso-2,3-BD from glucose, a sequential cultivation process with *Serratia marcescens*—a highly efficient meso-2,3-BD producer—was employed, resulting in 10 g/L 2-butanol [17]. Overexpression of the endogenous alcohol dehydrogenase *pduQ* in *L. diolivorans* further increased the final titer to 13.4 g/L, representing a 34% improvement. However, the co-cultures combining efficient 2,3-BD producers with whole-cell dehydratase catalysts require long fermentation times, low substrate concentrations, and complex process control, which can limit their industrial application [18,19].

From a biochemical standpoint, microbial production of 2-butanol relies on two enzymes: diol dehydratase, which converts meso-2,3-BD into 2-butanone, and alcohol dehydrogenase, which reduces 2-butanone to 2-butanol [20]. Metabolic engineering strategies targeting these steps have achieved the most promising results to date. An engineered *Klebsiella pneumoniae* strain expressing heterologous enzymes reached the highest known 2-butanol levels in single-strain systems [21]. However, these levels remain modest, and the full potential of *Klebsiella*-based platforms has yet to be realized.

*K. pneumoniae* G31 is a promising candidate to host the microbial 2-butanol production for several reasons. First, it is highly efficient at producing meso-2,3-BD from sugars and glycerol, generating a substantial internal supply of the immediate precursor needed for 2-butanol biosynthesis [22]. Second, *K. pneumoniae* exhibits relatively high tolerance to 2-butanol compared to many other facultative anaerobes, making it a suitable host for producing secondary alcohols [23]. Third, the species has natural genetic and biochemical traits that support diol dehydration reactions. Its genome contains a *pdu* operon encoding a B_12_-dependent diol dehydratase complex (*pduCDE*) and associated reactivation factors (*pduGH*), which maintain enzyme activity during catalysis. These enzymes are involved in the processing of 1,2-propanediol and related substrates; being often present in bacterial microcompartments that reduce the toxicity of reactive aldehyde intermediates [24].

Based on these considerations, we hypothesized that a heterologous diol dehydratase with improved specificity for meso-2,3-BD could efficiently redirect the native 2,3-BD pool toward 2-butanone formation in *K. pneumoniae*. *L. diolivorans* DSM 14421 was selected as the diol dehydratase source given its well-characterized *pduCDEGH* system and demonstrated ability to catalyze 2,3-BD dehydration [25,26]. In addition, alcohol dehydrogenases from *Clostridium* species have been reported to exhibit superior activity toward secondary ketones and alcohols compared to native *Klebsiella* enzymes, making them suitable candidates for catalyzing the terminal reduction step [27].

Despite increasing interest in microbial 2-butanol production, most reported strategies rely on multistep or multi-organism systems and often require external supplementation of pathway intermediates, limiting process integration and sustainability. In particular, a direct glucose-to-2-butanol route implemented within a single microbial host that naturally overproduces meso-2,3-BD remains insufficiently explored. Moreover, the relative impact of promoter architecture and enzyme choice on pathway performance in hosts such as *K. pneumoniae* has not been systematically evaluated. These limitations highlight the need for an integrated, host-adapted biosynthetic strategy enabling efficient and tunable 2-butanol production from glucose. Therefore, the aim of this study was to combine the high intrinsic meso-2,3-BD production capacity of *K. pneumoniae* G31 with heterologous diol dehydratase and alcohol dehydrogenase and to evaluate how promoter architecture influences pathway performance. This integrative strategy was designed to establish a functional and tunable glucose-to-2-butanol pathway within a single microbial host.

## 2. Results

### 2.1. Construction of a Synthetic 2-Butanol Pathway in Klebsiella pneumoniae

To enable direct microbial conversion of glucose into 2-butanol, a synthetic pathway was engineered by combining native and heterologous enzymatic modules in the *K. pneumoniae* strain G31. The approach was to extend the existing meso-2,3-BD pathway toward 2-butanone and 2-butanol.

**Figure 1:**
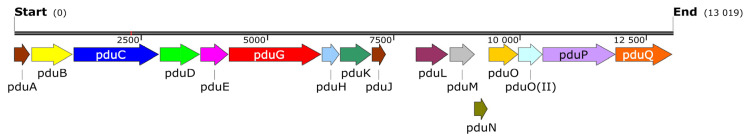
The *pdu* operon (13,019 bp) in *L. diolivorans* DSM 14421.

The propanediol utilization (*pdu*) operon in *L. diolivorans* DSM 14421 (Figure 1) is approximately 13 kb in length and contains proteins essential for the formation of bacterial microcompartments (BMC). The glycerol dehydratase operon comprises three genes: *pduC* (1677 bp), *pduD* (798 bp), and *pduE* (543 bp). It is followed by two genes encoding subunits of the enzyme reactivase: *pduG* (1830 bp) and *pduH* (351 bp).

Therefore, the diol dehydratase complex, encoded by *pduCDEGH* (5.8 kb), was heterologously expressed in *K. pneumoniae* G31 alongside either the native alcohol dehydrogenase *pduQ* from *L. diolivorans* DSM 14421 (1.1 kb) or an NAD(P)-dependent alcohol dehydrogenase (*adh*) from *C. beijerinc*kii (1.2 kb).

A series of recombinant constructs using the pCR^®^2.1-TOPO^®^ backbone was created to analyze the roles of these individual pathway components and to assess how promoter strength affects pathway performance.

Table S1 summarizes the recombinant plasmids obtained, including gene composition, promoter setup, and plasmid size.

**Figure 2:**
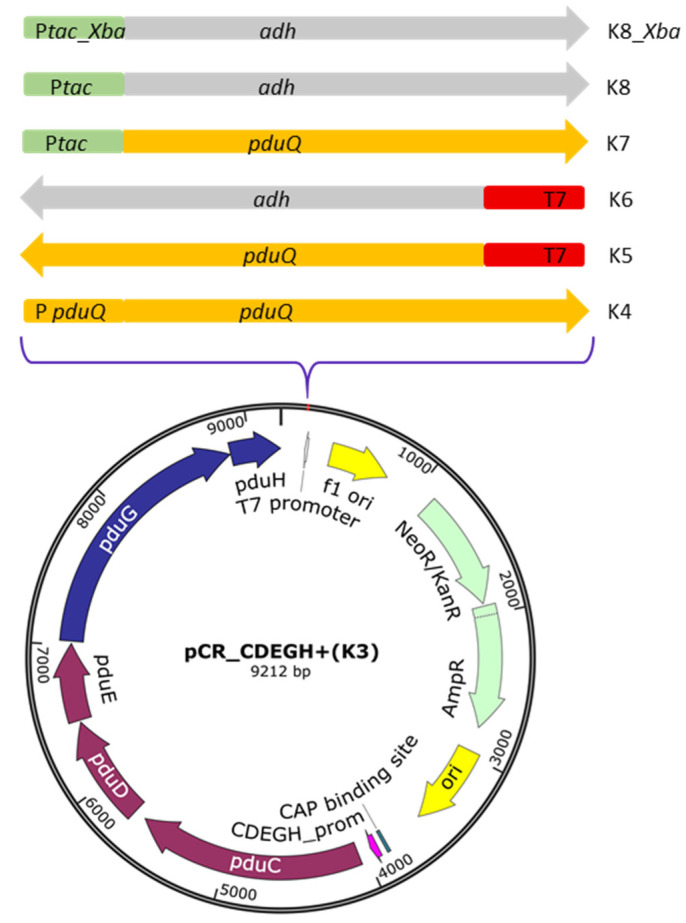
Recombinant constructs derived from pCR_*pduCDEGH*+ under the control of the native promoter of *pduC*.

While the *pduCDEGH* operon and *pduQ* were expressed under their native promoters (Figure 2), *pduQ* was also cloned downstream of the strong T7 and P*tac* promoters to enhance expression levels, as was similarly performed for the *adh* gene.

### 2.2. Baseline Metabolism of Klebsiella pneumoniae G31 and Comparison with a Reference Strain

Before pathway engineering, the basic metabolic profiles of *K. pneumoniae* G31 and the reference strain *K. pneumoniae* ATCC 9621 were analyzed and compared. During batch fermentation in medium containing 60 g/L glucose under anaerobic conditions, both *K. pneumoniae* G31 and ATCC 9621 efficiently converted glucose to meso-2,3-BD, along with traces of 2-butanone (17–18 mg/L) and 2-butanol (<15 mg/L). These results indicate that native strains of *K. pneumoniae* may be capable of producing small amounts of 2-butanol directly from glucose. However, the concentrations detected, especially for 2-butanol, were very low (close to the limits of detection) and were obtained with high statistical error (Table 1).

### 2.3. Effect of Heterologous Genes on 2-Butanol Formation by Engineered K. pneumoniae Strains

To evaluate the impact of heterologous diol dehydration and reduction on pathway performance, the formation of 2-butanone and its subsequent conversion to 2-butanol were analyzed in engineered *K. pneumoniae* G31 derivatives cultivated anaerobically for 120 h on 60 g/L glucose as a sole carbon source (Table 1).

Introducing the *pduCDEGH* operon alone (strain K3) resulted in the accumulation of 69 mg/L 2-butanone and 110 mg/L 2-butanol. This indicates not only the functional expression of the heterologous diol dehydratase from *L. diolivorans* in the *Klebsiella* host but also the action of the native alcohol dehydrogenase of the host.

Co-expression of *pduQ* (strains K4, K5, and K7) yielded 2-butanone concentrations ranging from 71 to 119 mg/L, accompanied by relatively low 2-butanol titers (89–124 mg/L). These results indicate that although *pduQ* enables the reduction of 2-butanone, it does not provide sufficient catalytic capacity to substantially increase 2-butanol formation under the tested conditions.

In contrast, introducing a heterologous alcohol dehydrogenase from *C. beijerinckii* significantly altered the pathway output. Strain K6, which carries T7-driven *adh*, showed a clear shift toward 2-butanol production, reaching 437 ± 32 mg/L. This was the highest titer among all engineered strains, representing a 40-fold increase over the parental G31. This increase was accompanied by evidently lower residual meso-2,3-BD levels, indicating improved downstream flow to 2-butanol and higher concentrations of lactic acid and ethanol at the end of the process. Strains expressing *adh* under the control of the P*tac* promoter (K8 variants) showed intermediate 2-butanol titers (191–201 mg/L). Compared with the *K. pneumoniae* G31-derived constructs, the corresponding K6 and K8 derivatives in ATCC 9621 as the host showed significantly lower 2-butanol titers despite having comparable or higher residual meso-2,3-BD levels, indicating inefficient coupling between diol dehydration and terminal reduction.

### 2.4. Conversion of Exogenously Supplied 2-Butanone to 2-Butanol by Engineered K. pneumoniae

To evaluate the independent reductive step of the engineered pathway, separate from diol dehydration, the ability of recombinant *K. pneumoniae* strains to convert externally supplied 2-butanone into 2-butanol was tested. Cultures were grown in a medium containing 10 g/L 2-butanone and 5 g/L glucose, and metabolite profiles were analyzed after 120 h of incubation. Both wild-type strains, *K. pneumoniae* G31 and ATCC 9621, efficiently converted 2-butanone to 2-butanol, producing 2.5 g/L and 2.6 g/L, respectively. These results show that native *Klebsiella* alcohol dehydrogenases exhibit strong activity toward 2-butanone when the substrate is present, even though they cannot efficiently produce 2-butanol from glucose alone. All engineered strains successfully reduced externally supplied 2-butanone to 2-butanol, confirming that alcohol dehydrogenase activity is generally sufficient when the substrate is readily available. Genetically modified strains that contained *pduQ* from *L. diolivorans* produced lower titers of 2-butanol compared to wild-type strains. The highest titers were still observed in strains with *adh*: strain K6 (T7-driven expression) reached 3.9 g/L, and K8 (P*tac*-driven expression) reached 3.0 g/L (Table 2). Clone K6 showed the highest conversion rate of 2-butanone to 2-butanol, with a yield of 0.85 g/g.

### 2.5. Quantitative Analysis of the pduC, pduQ, and adh Gene Expression

**Figure 3:**
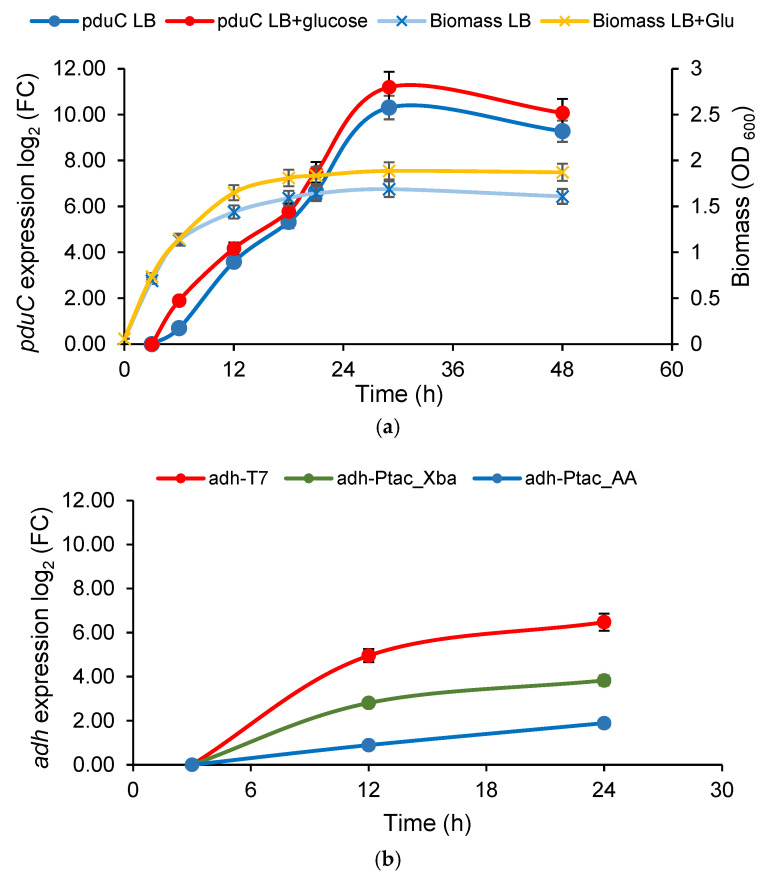
(**a**) Expression profile of *pduC* under control of its native promoter in *K. pneumoniae* G31, containing *pduCDEGH* and *adh* (K6) during batch cultivation in LB medium, or LB with 20 g/L glucose, at aerobic conditions. FC, fold change; (**b**) effect of the promoters on *adh* transcription levels in engineered *K. pneumoniae* G31 (K6, K8_P*tac*_AA, and K8-P*tac*_Xba) as measured by RT-qPCR. FC indicates fold change. Cultivation was carried out in LB medium with 20 g/L glucose under aerobic conditions.

Quantitative gene expression analysis by RT-qPCR was conducted to assess the transcriptional activity of the engineered pathway and the influence of promoter selection. When *pduC* was expressed under its native promoter, all recombinant constructs achieved comparable transcript levels. Construct K6, as the best 2-butanol producer, was chosen as a representative for detailed temporal analysis (Figure 3a).

As shown in Figure 3, *pduC* transcription in K6 remained low during early exponential growth and increased sharply after about 20 h of cultivation, reaching peak levels at the 29th hour. These dynamics closely followed biomass accumulation and were further enhanced in the presence of glucose, indicating that the native *pduC* promoter is regulated in a growth- and substrate-dependent manner. After reaching peak expression, *pduC* transcript levels gradually declined. To assess promoter-dependent transcriptional differences independently of product formation, relative transcript levels of *pduQ* and *adh* under native, P*tac*, and T7 promoters were quantified by RT-qPCR (Figure 3b). Replacement of the native *pduQ* promoter with P*tac* or T7 resulted in a moderate but consistent increase in transcript levels (1.5-fold and 2.2-fold, respectively), confirming transcriptional differences between promoter configurations. In contrast, *adh* expression showed a strong dependence on promoter selection, as reflected by significantly elevated transcript levels under T7 control. P*tac*-driven *adh* constructs (K8) showed a 22-fold increase in transcript levels at 24 h compared to 3 h of cultivation, while T7-driven constructs (K6) exhibited an over 110-fold increase over the same time period. Notably, adding an eight-nucleotide 5′ non-translated sequence (5′-TCTAGAAA-3′) between the ribosome-binding site and the start codon (P*tac*-Xba) further increased *adh* transcription, resulting in more than a sixfold increase over constructs without this modification (P*tac*-AA).

In standard cloning and expression systems, the T7 promoter is typically paired with the phage-encoded T7 RNA polymerase, which is absent from the genomic background of strain G31. Despite this, strong gene expression from T7-based constructs was consistently observed. To address this discrepancy, transcriptional activity was analyzed using rifampicin, which specifically inhibits bacterial host RNA polymerase but does not affect phage-derived enzymes. Treatment with 200 μg/mL rifampicin for 30 min during early exponential growth immediately halted cellular growth, with the optical density (OD_600_) remaining stable at approximately 0.3 in the treated culture, compared to approximately 0.6 in the untreated control. RT-qPCR analysis showed that rifampicin reduced adh transcript levels to approximately 4% of control levels, consistent with the absence of phage RNA polymerase activity. A nearly 6-fold decrease in *rpoD* transcript levels further confirmed effective inhibition of bacterial RNA polymerase. Gene expression levels were normalized to the internal control 16S rRNA, which was largely unaffected by rifampicin. No T7 RNA polymerase-dependent transcription was detected under these conditions. Expression from constructs containing the T7 promoter was observed in the absence of phage polymerase, indicating transcription by the native *K. pneumoniae* G31 RNA polymerase.

### 2.6. Structural Features of Heterologous Enzymes Relevant to Pathway Performance

**Figure 4:**
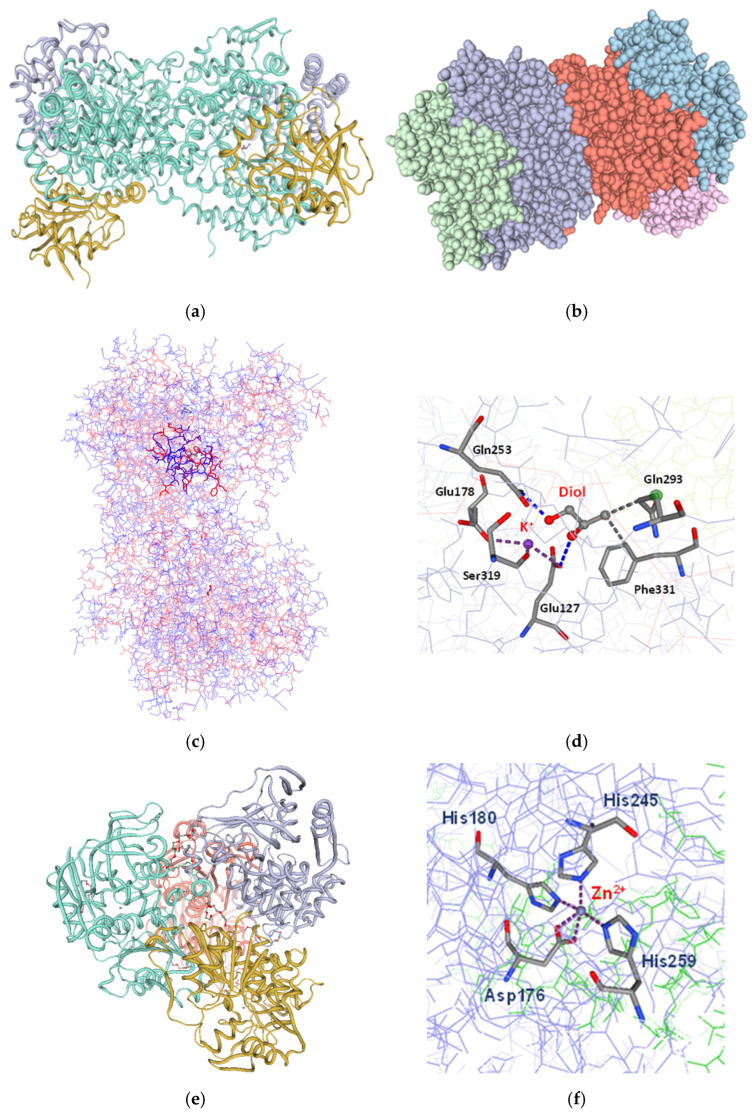
Three-dimensional models of *L. diolivorans* diol dehydratase and *C. beijerinkii* DSM 51 alcohol dehydrogenase. (**a**) Hetero-2-2-2-mer oligomeric state; cyan chains, PduC; yellow chains, PduD; purple chains, PduE; (**b**) spacefill model; (**c**) licorice model showing the active site; (**d**) coordination of the substrate and potassium ion; (**e**) the homotetrameric structure of Adh of *C. beijerinkii* DSM 51 with chains shown in different colors; (**f**) coordination of the Zn^2+^. Purple dashed lines indicate interactions in the metal complex; blue and gray dashed lines represent hydrophobic interactions. The models were created in the SWISS-Model workspace [27]; the Adh comparison used the crystal structure reported by Korkhin et al. [28].

Three-dimensional models of the heterologous diol dehydratase from *L. diolivorans* and the alcohol dehydrogenase (Adh) from *C. beijerinckii* were created using the SWISS-Model workspace [15]. Structural analysis of PduCDE showed a hetero-2-2-2-mer oligomeric organization composed of the PduC, PduD, and PduE subunits (Figure 4), consistent with typical B_12_-dependent diol dehydratases.

A detailed inspection of the active site revealed conserved cofactor- and metal-binding motifs essential for substrate binding and catalysis (Figure 4b–d). Substrate activation is facilitated by a network of conserved polar residues, with Glu170, Glu247, and Asp335 playing key roles in hydrogen-bonding and polarization of the diol hydroxyl groups, thereby aiding hydroxyl migration and water elimination. Two K^+^ ions per oligomer were identified as crucial structural elements that stabilize the active-site architecture rather than directly participating in catalysis. Potassium coordination involves residues E127, E178, and S319, with E127 and E178 contributing to both metal binding and substrate positioning. Additional hydrogen-bonding interactions with E127 and Q253 ensure correct substrate orientation, while hydrophobic contacts, especially involving F331, limit substrate mobility and support efficient catalysis.

Sequence analysis of the *adh* gene of *C. beijerinckii* DSM 51 revealed that it encodes a 351-amino-acid NADP-dependent alcohol dehydrogenase, similar to that of *C. beijerinckii* strain NRRL B593, with seven amino acid substitutions, none of which are in the active site. Structural modeling of the enzyme predicted a homotetrameric assembly, with each subunit coordinating one Zn^2+^ ion and one NADPH molecule (Figure 4e). The zinc ion is non-covalently bound within the active site and coordinated by conserved residues, forming a stable metal complex essential for catalytic activity (Figure 4f). The close spatial arrangement of Zn^2+^, NADPH, and the substrate-binding pocket supports efficient hydride transfer during ketone reduction.

These structural features suggest that optimal pathway performance in vivo depends on the availability of key cofactors and metal ions, including coenzyme B_12_, K^+^, Zn^2+^, and NADPH. Therefore, optimizing the medium composition is critical in maximizing the efficiency of diol dehydration and terminal reduction in the engineered glucose-to-2-butanol pathway.

## 3. Discussion

Microbial production of 2-butanol has long been considered an attractive yet elusive goal in industrial biotechnology. Although it offers better fuel properties and lower toxicity than 1-butanol, microbial titers reported to date remain modest, even though metabolic engineering of these enzymatic activities in *Klebsiella* spp. has achieved some of the highest 2-butanol titers reported to date. For example, *K. pneumoniae* HR526 produced 1.03 g/L of 2-butanol after heterologous expression of a diol dehydratase from *L. brevis* (encoded by *pduCDEGH*) and an alcohol dehydrogenase (*adh*) from *C. autoethanogenum*, combined with the deletion of the lactate dehydrogenase gene (*ldhA*), the introduction of a point mutation in the diol dehydratase active site, and supplementing the medium with 1 µM coenzyme B_12_ [16]. In another study, heterologous expression of alcohol dehydrogenase (*adhA*) and α-ketoisovalerate decarboxylase (*kivD*) from *Lactococcus lactis* in *K. pneumoniae*, along with the disruption of competing pathways that lead to lactate and 2,3-BD, resulted in the production of 320 mg/L of isobutanol from crude glycerol [29,30].

Further, we demonstrate that combining natural metabolic potential with selected heterologous enzymes enables conversion of glucose to 2-butanol within a single microbial host, providing the first data on the expression of diol dehydratase from *L. diolivorans* and the *adh* gene from *C. beijerinckii* DSM 51 in *K. pneumoniae*.

A key part of this strategy was selecting *K. pneumoniae* G31 as the production host. Unlike common laboratory strains, G31 is an established overproducer of meso-2,3-BD under non-engineered conditions, providing a high internal substrate supply for downstream conversion [23,31,32]. Since 2,3-BD availability is the main entry point into the 2-butanol pathway, this naturally occurring trait significantly reduces the need for extensive upstream metabolic engineering. Beyond its advantageous metabolite profile, *K. pneumoniae* possesses inherent genetic traits that facilitate diol dehydration reactions. The presence of a native *pdu* operon encoding a B_12_-dependent diol dehydratase (*pduCDE*) and associated reactivation factors (*pduGH*) [33] indicates that this organism is naturally equipped to manage radical-based catalysis and potentially toxic aldehyde intermediates [34]. Although the endogenous *pdu* system primarily functions in 1,2-propanediol metabolism, its presence suggests it has the capacity to support diol dehydration chemistry more broadly, including cofactor regulation and possibly spatial organization within bacterial microcompartments [35]. This native system likely minimizes regulatory conflicts and metabolic stress upon heterologous expression of B_12_-dependent diol dehydratases, thereby reducing the metabolic and regulatory challenges associated with introducing heterologous enzymes.

In this context, *L. diolivorans* DSM 14421 was selected as a source of heterologous *pduCDEGH* genes. Although most authors assert that its *pdu*-encoded diol dehydratase system is mainly specialized for 1,2-propanediol [36,37,38], Russmayer et al. (2019) [17] showed that the enzymatic machinery in *L. diolivorans* is also highly effective at converting meso-2,3-BD into 2-butanone.

While efficient diol dehydration is crucial to initiate the pathway toward 2-butanol, the subsequent reduction of 2-butanone to 2-butanol is another key step. In our study, we evaluated different alcohol dehydrogenase genes and promoters. Strains K4, K5, and K7 expressed *pduQ* from *L. diolivorans* under its native promoter, the T7 promoter, or the P*tac* promoter, respectively. Despite effective diol dehydration, all three strains showed consistently low 2-butanol levels, indicating that PduQ alone is insufficient to support the reduction of 2-butanone in the *Klebsiella* host. Notably, increasing transcriptional strength via T7 or P*tac* did not significantly improve production using the *pduQ* gene, suggesting that inherent structural limitations, rather than expression levels, restrict enzyme performance in vivo. In contrast, alcohol dehydrogenases from *Clostridium* species are known to have a superior activity toward secondary ketones and alcohols [39]. Consistent with this trait, heterologous expression of a clostridial NADP-dependent alcohol dehydrogenase significantly increased 2-butanol production in *K. pneumoniae* G31.

Interestingly, our results indicated that native *K. pneumoniae* also expresses strong secondary alcohol dehydrogenases, as both wild-type strains efficiently converted 2-butanone to 2-butanol, producing up to 2.5–2.6 g/L of the alcohol. Such activity is consistent with the presence of native enzymes involved in 2,3-butanediol metabolism, particularly the acetoin reductase (BudC), which belongs to the short-chain dehydrogenase/reductase family and can exhibit activity toward structurally related ketones [40,41]. Moreover, strain K3 produced ~110 mg/L of 2-butanol, despite lacking any heterologously expressed alcohol dehydrogenase capable of catalyzing this reaction.

Systematic adjustment of *adh* expression further demonstrated that the choice of promoter significantly influences pathway output. The recombinant strain K6, which uses T7-driven *adh* expression, consistently outperformed the P*tac*-driven K8 variants, as indicated by higher 2-butanol titers. The highest conversion of butanone was observed in strain K6, where 3.9 g/L of 2-butanol was produced, while the P*tac*-controlled K8 strain yielded a comparatively lower titer of 3.0 g/L. These results demonstrate that diol dehydration and butanone reduction impose different and sequential constraints on pathway flux. While effectively expressing *pduCDEGH* overcomes the initial dehydration bottleneck, the overall success of the pathway heavily depends on the type and level of expression of the terminal alcohol dehydrogenase. The consistently poor performance of *pduQ* across promoter configurations highlights the enzyme’s limitations in the engineered *K. pneumoniae* system, whereas consistently high expression of a clostridial *adh* under T7 control enables more efficient 2-butanone reduction. These findings reveal that promoter strength alone cannot compensate for suboptimal enzyme properties and emphasize the importance of selecting appropriate enzymes, expression strategies, and host physiology when designing non-native alcohol biosynthesis pathways.

The selected promoter sequence, derived from the canonical T7 promoter but harboring mutations and a fused translation initiation region, supports sustained high levels of expression driven by the endogenous *K. pneumoniae* RNA polymerase, consistent with efficient recognition of its −10/−35-like elements by the host σ-dependent transcription machinery. Sequence analysis of the region upstream of *adh* in K6-construct reveals that the canonical T7 core promoter contains AT-rich motifs (TAATAC) that resemble the σ^70^ −10 consensus sequence (TATAAT). Although the match is not perfect, σ^70^ is known to tolerate significant deviations from consensus, especially when the downstream context is favorable [42].

**Figure 5:**
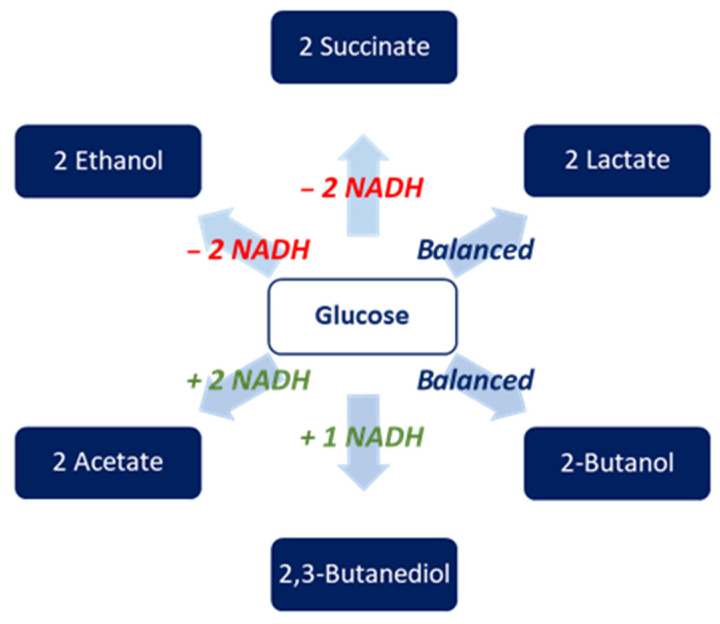
NADH balance in the formation of fermentation products from 1 mol glucose in engineered *K. pneumoniae*.

Redox balance is a key factor influencing pathway efficiency. The reduction of 2-butanone to 2-butanol depends entirely on NADH or NADPH, and competition for these reducing agents is likely, since 2,3-BD synthesis itself acts as an NADH consumer in *K. pneumoniae*. Converting glucose to 2-butanol in a genetically engineered *K. pneumoniae* strain involves a complex balance of cofactors. During glycolysis, one mole of glucose produces two moles of pyruvate and gains 2 NADH. The subsequent production of 2,3-BD from two pyruvate molecules uses 1 NADH at the acetoin reductase step, creating a temporary excess of 1 NADH. The heterologous pathway then converts 2,3-BD to 2-butanone, finally reduced to 2-butanol. The last step requires 1 NADH (or 1 NADPH); therefore, redox availability, rather than substrate supply, becomes limiting at higher expression levels. For example, when 2-butanone was used as the substrate, both engineered and native *K. pneumoniae* strains increased their 2-butanol production further due to higher NADH availability. In fact, the major co-product in these processes appeared to be acetic acid, a metabolic product in which NADH is not consumed but is released (Figure 5).

This demonstrates that pathway engineering specifically enhances terminal 2-butanone reduction without notably disrupting central carbon flux. Although *K. pneumoniae* ATCC 9621 efficiently reduces 2-butanone when supplied externally, its strong tendency to produce extracellular polysaccharides creates a competing pathway that diverts carbon flux and reducing equivalents away from 2-butanol biosynthesis [36]. This limitation further hampers its ability to support an integrated, glucose-driven 2-butanol pathway compared to the G31 background. The G31 host background clearly offers a more favorable metabolic and redox environment for directing carbon flux toward 2-butanol production.

Beyond redox balance, the energy efficiency of 2-butanol production in *K. pneumoniae* remains positive, providing a net gain of 2 ATP per mole of glucose through substrate-level phosphorylation during glycolysis. However, the diol dehydratase system (PduCDE) depends on coenzyme B_12_ (adenosylcobalamin), which is often inactivated during catalysis. Recharging this cofactor with its specific reactivase (PduGH) requires an ATP-dependent step, potentially causing a small energy drain. Despite this, the overall pathway is energetically self-sufficient, generating sufficient ATP surplus to support both cell growth and the metabolic demands of heterologous protein expression, even in the absence of oxygen.

Compared to previously reported methods for microbial synthesis of 2-butanol, this study establishes *K. pneumoniae* G31 as a promising platform for microbial 2-butanol production through rational pathway engineering. Our findings highlight the importance of aligning enzyme specificity, promoter activity, and host redox balance, and demonstrate that non-canonical σ-factor-dependent promoter activity can create an optimal expression environment. These insights provide a solid foundation for further strain development and process improvements to achieve industrially relevant titers of 2-butanol.

## 4. Materials and Methods

### 4.1. Strains, Media, and Culture Conditions

*L. diolivorans* DSM 14421 was purchased from the German Collection of Microorganisms and Cell Cultures GmbH (Deutsche Sammlung von Mikroorganismen und Zellkulturen, DSMZ, Braunschweig, Germany). *K. pneumoniae* G31 was isolated from the active slime of wastewater treatment and is stored in NBIMCC under the number 8645 [17]. *Escherichia coli* HST08 strain STELLAR^TM^ competent cells were purchased from Clontech Laboratories Inc., Takara Bio Company (Mountain View, CA, USA), as shown in Table 3.

*K. pneumoniae* strains were cultivated in a selected working medium with the following composition (g/L): glucose, 60; yeast extract, 5; CH_3_COONa × 3H_2_O, 3; FeSO_4_ × 7H_2_O), 0.02; MnSO_4_ × H_2_O, 0.0115; ZnSO_4_ × 7H_2_O, 0.001; (NH_4_)_2_HPO_4_, 3; MgSO_4_ × 7H_2_O, 0.6; KCl, 4; CaCO_3_, 30. Coenzyme B_12_ (1 µM) and kanamycin (50 µg/mL) were added freshly. *L. diolivorans* DSM 14421 was cultured in MRS medium under anaerobic conditions, AnaeroGen^TM^ (Thermo Scientific Inc., Waltham, MA, USA).

For inoculum preparation, *Klebsiella* strains were cultured in 100 mL flasks containing 50 mL of LB supplemented with 20 g/L of glucose overnight at 37 °C and 180 rpm in a shaking incubator (Model SKI 4, Argo Lab, Carpi, Italy). For 2-butanol production, a 2% inoculum was added to 100 mL of working medium, which was cultured in laboratory bottles (Boeco, Hamburg, Germany) in a jar under an anaerobic atmosphere generated by AnaeroGenTM packs, with shaking at 180 rpm. Kanamycin (50 µg/mL) and coenzyme B12 (1 µM) were added fresh before cultivation.

### 4.2. Nucleic Acid Isolation and PCR Amplification Conditions

Total DNA and RNA from samples collected at different hours were extracted using a Gene-MATRIX Bacterial and Yeast Genomic DNA Purification Kit and a GeneMATRIX Universal Purification Kit, respectively, following the manufacturer’s instructions (EURx, Gdansk, Poland), including on-column DNase I treatment for RNA.

PCR amplification of all vectors and genes was carried out using TaKaRa Taq Version 2.0 (Clontech Laboratories Inc., a Takara Bio company, Mountain View, CA, USA) for fragments up to 4 kb, and Q5^®^ Hot Start High-Fidelity 2× Master Mix (New England Biolabs, Ipswich, MA, USA) for fragments larger than 4 kb. The reaction volume was 25 μL.

PCR conditions and primers are listed in Table 4. PCR products were visualized on a 1% agarose gel stained with the fluorescent dye SimplySafe (EURx, Gdansk, Poland).

The NEBuilder^®^ HiFi DNA Assembly Master Mix (New England Biolabs, Ipswich, MA, USA), which uses a Gibson-type isothermal assembly method, was employed for cloning. Assembly reactions were performed in a final volume of 10 µL, typically including 200 ng of linearized vector and equal molar amounts of insert DNA. The mixtures were incubated at 50 °C for 60 min, with the heated lid kept at 60 °C to prevent condensation.

All PCR and cloning assembly reactions were incubated in a MultiGene OptiMax Thermal Cycler (Labnet International, Inc., Edison, NJ, USA). Sequencing was performed by Macrogen Inc. (Amsterdam, The Netherlands).

### 4.3. Reverse Transcription and Real-Time qPCR

Reverse transcription (RT) was performed using the NG dART RT Mix (EURx, Gdansk, Poland). Reactions of 20 μL included 1 μg of total RNA and 200 ng of random hexamer primers and were performed as follows: 10 min at 25 °C for primer hybridization, 50 min at 50 °C for reverse transcription, and 5 min at 85 °C to inactivate the enzyme.

Real-time RT-qPCR analyses were conducted using iTaq Universal SYBR Green Supermix on a CFX96 Touch Real-Time PCR Detection System (Bio-Rad, Hercules, CA, USA). Reactions were performed in a final volume of 20 µL, usually containing 40 ng of cDNA template and 500 nM of each primer. Primer pairs were designed to produce approximately 100 bp amplicons (Table 5).

All amplification reactions were carried out at 60 °C. Relative gene expression levels were calculated using the comparative ΔΔCt method. Ct values of the target genes were normalized to the *rpoD* reference gene. All RT-qPCR experiments were performed with three biological replicates, each analyzed in technical triplicate. Expression values were determined as arithmetic means, and data are presented as mean ± standard deviation.

### 4.4. Construction of Recombinant Strains

The *pduCDEGH* operon and *the pduQ* gene from *L. diolivorans* DSM 14421 (Table 6) were amplified using gene-specific primers (Table 7) designed from NCBI-GenBank sequences.

Amplification of the alcohol dehydrogenase gene (*adh*) from *C. beijerinckii* DSM 51 was initially based on the sequence of its closest known homolog, the *adh* gene of *C. beijerinckii* NRRL B593 (GenBank accession no. AF157307), [45]. The amplified *adh* gene from DSM 51 was subsequently sequenced and deposited in GenBank.

### 4.5. Transformation of E. coli and K. pneumoniae

Transformation of *E. coli* STELLAR^TM^-competent cells was performed according to the manufacturer’s protocol PT5055-2, with some modifications. Briefly, 50 μL of cells were thawed on ice, mixed with the construct to be transformed, incubated on ice for 30 min, heat-shocked for 45 s at 42 °C, and incubated on ice for 1–2 min. Warmed SOC medium was added to reach a final volume of 500 μL, and the cells were incubated for 1 h at 37 °C with shaking at 160 rpm. The cells were then plated on standard Petri dishes containing 50 μg/mL kanamycin to select transformants.

*K. pneumoniae* wild-type strains were transformed via electroporation using a pulse of 1.8 kV for 4.5 to 5.5 ms in cuvettes with a 0.1 cm electrode gap on a MicroPulser electroporator (BioRad Laboratories, Hercules, CA, USA). Electrocompetent *K. pneumoniae* cells were prepared from fresh overnight cultures grown in low-salt LB medium (1% tryptone, 0.5% yeast extract, 0.05% NaCl) to an OD600 of 0.5–0.6. After three washes with ice-cold 1 mM HEPES, sterile water, and 10% glycerol, the cells were resuspended in 1/100 of the initial culture volume in 10% glycerol and stored at –70 °C.

### 4.6. Rifampicin Treatment

*K. pneumoniae* cultures were treated with rifampicin, as described previously [46,47]. Early exponential phase cultures with an OD_600_ of approximately 0.3–0.4 were supplemented with 200 μg/mL rifampicin and incubated for 30 min before sampling. Growth was monitored by OD_600_ measurements, and cells were collected for RNA extraction. RT-qPCR was used to measure target gene expression relative to untreated controls, with 16S rRNA serving as an internal control.

### 4.7. Analytical Methods

Sugars and fermentation products were quantified using a YL Instrument 9300 HPLC System (YL Instrument Co., Ltd., Anyang, South Korea). Separation was performed on an Aminex HPX-87H column (BioRad Laboratories, Hercules, CA, USA) maintained at 65 °C, using 5 mM H_2_SO_4_ as the mobile phase at a flow rate of 0.6 mL/min. Glucose, meso-2,3-BD, L-2,3-BD, 2-butanone, and 2-butanol were detected using a refractive index (RI) detector (YL 9170 RI Detector), while succinic acid, lactic acid, and acetic acid were monitored with a UV/Vis detector (YL 9120 UV/Vis Detector) at wavelengths of 210 nm.

## 5. Conclusions

This study demonstrates the direct microbial conversion of glucose to 2-butanol by extending the native meso-2,3-BD pathway in *K. pneumoniae* G31. By combining the host’s natural ability to produce 2,3-BD with the heterologous expression of a B_12_-dependent diol dehydratase system from *L. diolivorans* and an effective alcohol dehydrogenase from *C. beijerinckii*, a functional biosynthesis route to 2-butanol was established. The results identify 2,3-BD dehydration as the main bottleneck in the glucose conversion process. Expression of *adh* under the T7 promoter achieved the most efficient conversion, producing the highest amount of 2-butanol from both glucose and 2-butanone substrates. Future research should focus on enhancing the expression of the *L. diolivorans* diol dehydratase and optimizing its amino acid sequence to increase its affinity for the substrate 2,3-BD, as our experiments showed good agreement between the host *K. pneumoniae* and this heterologous enzyme in terms of physiological and biochemical performance. Another key aspect of improving the biotechnological process should involve transferring the optimized pathway to a stable, chromosomally integrated expression system and further optimizing culture conditions, including medium composition and oxygen supply, to enhance the sustainability, scalability, and industrial relevance of 2-butanol production.

## Figures and Tables

**Table 1:** Products obtained from glucose as a sole carbon source by native and engineered *K. pneumoniae* strains.

Strain	Products									
	SA (g/L)	LA (g/L)	AA (g/L)	meso-2,3-BD (g/L)	L-2,3-BD (g/L)	EtOH (g/L)	2-Butanone (mg/L)	2-Butanol (mg/L)	2-Butanol (mg/L/h)	2-Butanol (mg/g) ^1^
**Native strains**										
G31	0.9 ± 0.1	2.2 ± 0.2	0.5 ± 0.1	13.9 ± 0.2	3.7 ± 0.3	9.0 ± 0.1	17 ± 5	11 ± 5 ^2^	0.09	0.18
ATCC 9621	0.7 ± 0.1	2.3 ± 0.3	0.4 ± 0.1	13.6 ± 0.2	3.6 ± 0.4	8.7 ± 0.3	18 ± 6	14 ± 5 ^2^	0.12	0.23
**Engineered strains**										
G31 K3	1.0 ± 0.1	4.1 ± 1.0	0.7 ± 0.2	12.5 ± 0.2	3.8 ± 0.4	8.7 ± 0.4	69 ± 8	110 ± 4	0.92	1.83
G31 K4	0.9 ± 0.0	2.4 ± 0.6	0.5 ± 0.0	12.7 ± 0.2	4.0 ± 0.0	9.1 ± 0.1	73 ± 6	108 ± 5	0.90	1.80
G31 K5	0.8 ± 0.1	1.7 ± 0.4	0.4 ± 0.1	13.5 ± 1.6	4.3 ± 0.1	9.2 ± 0.2	71 ± 25	89 ± 19	0.74	1.48
G31 K6	1.2 ± 0.0	7.4 ± 0.3	0.9 ± 0.0	6.5 ± 0.8	6.5 ± 0.6	10.3 ± 0.3	109 ± 28	437 ± 32	3.64	7.28
G31 K7	0.9 ± 0.2	2.9 ± 0.2	0.5 ± 0.1	12.6 ± 1.9	4.4 ± 0.2	9.2 ± 0.3	119 ± 38	124 ± 21	1.03	2.07
G31 K8 ^3^	1.0 ± 0.1	7.3 ± 0.3	1.1 ± 0.0	10.6 ± 1.8	2.8 ± 0.2	7.7 ± 0.4	95 ± 16	191 ± 18	1.59	3.18
G31 K8 ^4^	1.2 ± 0.1	7.8 ± 0.2	1.3 ± 0.0	10.4 ± 0.2	3.6 ± 0.1	8.5 ± 0.2	98 ± 18	201 ± 16	1.68	3.35
ATCC 9621 K6	1.3 ± 0.2	18.3 ± 0.4	1.3 ± 0.1	8.5 ± 0.2	0.7 ± 0.0	5.3 ± 0.1	127 ± 6	40 ± 4	0.33	0.69
ATCC 9621 K8	0.8 ± 0.1	5.1 ± 0.3	0.7 ± 0.0	14.3 ± 0.2	2.8 ± 0.0	8.8 ± 0.2	59 ± 8	38 ± 2	0.32	0.63

^1^ mg produced 2-butanol per gram glucose consumed; ^2^ supposed 2-butanol, the detected amounts are close to the detection level; ^3^ K8-*Ptac*_AA variant; ^4^ K8-P*tac*_Xba variant. The presented values were obtained after 120 h of cultivation in bottles in an anaerobic jar at 37 °C, shaken at 180 rpm. SA, succinic acid; LA, lactic acid; AA, acetic acid; 2,3-BD, 2,3-butanediol; EtOH, ethanol. All data represent the mean ± standard deviation of at least three independent biological replicates.

**Table 2:** Products obtained from medium containing 5 g/L glucose and 10 g/L 2-butanone as carbon sources by native and engineered *K. pneumoniae* strains.

Strain	Products									
	SA (g/L)	LA (g/L)	AA (g/L)	meso-2,3-BD (g/L)	L-2,3-BD (g/L)	EtOH (g/L)	2-Butanone (g/L)	2-Butanol (g/L)	2-Butanol (g/L/h)	2-Butanol (g/g) ^1^
**Native strains**										
G31	0.2 ± 0.0	0.2 ± 0.0	2.5 ± 0.1	0.7 ± 0.0	0.3 ± 0.0	0.7 ± 0.1	5.0 ± 0.4	2.5 ± 0.0	0.021	0.50
ATCC 9621	0.2 ± 0.0	0.3 ± 0.0	2.3 ± 0.1	0.7 ± 0.0	0.4 ± 0.0	0.4 ± 0.0	4.7 ± 0.2	2.6 ± 0.1	0.022	0.50
**Engineered strains**										
G31 K3	0.2 ± 0.0	0.2 ± 0.0	2.3 ± 0.0	0.7 ± 0.0	0.2 ± 0.0	0.9 ± 0.1	5.1 ± 0.1	2.2 ± 0.1	0.018	0.44
G31 K4	0.2 ± 0.0	0.2 ± 0.0	2.5 ± 0.1	0.8 ± 0.0	0.2 ± 0.0	0.6 ± 0.0	6.5 ± 0.1	2.1 ± 0.1	0.018	0.59
G31 K5	0.2 ± 0.0	0.2 ± 0.0	2.5 ± 0.1	0.8 ± 0.2	0.3 ± 0.1	0.6 ± 0.1	6.8 ± 0.3	2.1 ± 0.2	0.018	0.66
G31 K6	0.2 ± 0.0	0.2 ± 0.0	2.7 ± 0.0	0.7 ± 0.1	0.3 ± 0.0	0.5 ± 0.0	5.4 ± 0.2	3.9 ± 0.1	0.033	0.85
G31 K7	0.2 ± 0.0	0.2 ± 0.0	2.4 ± 0.2	0.9 ± 0.1	0.3 ± 0.0	0.7 ± 0.1	7.3 ± 0.3	2.2 ± 0.2	0.018	0.83
G31 K8 ^2^	0.2 ± 0.0	0.2 ± 0.0	2.6 ± 0.1	0.8 ± 0.1	0.3 ± 0.0	0.6 ± 0.0	6.2 ± 0.1	3.0 ± 0.0	0.025	0.77

^1^ Gram produced 2-butanol per gram 2-butanone consumed; ^2^ K8-P*tac*_Xba variant. The presented values were obtained after 120 h of cultivation in bottles in an anaerobic jar at 37 °C, shaken at 180 rpm. SA, succinic acid; LA, lactic acid; AA, acetic acid; 2,3-BD, 2,3-butanediol; EtOH, ethanol. All data represent the mean ± standard deviation of at least three independent biological replicates.

**Table 3:** Strains and constructs used in this study.

Strain	Description	Reference
*E. coli HST08* (Stellar^TM^)	F–, *endA1*, *supE44*, *thi-1*, *recA1*, *relA1*, *gyrA96*, *phoA*, *Φ80d* l*acZ*Δ M15, Δ(*lacZYA-argF*) U169, Δ(*mrr-hsdRMSmcrBC*), Δ*mcrA*, λ–	Clontech Laboratories Inc., Takara Bio
*L. diolivorans* DSM 14421	Source of *pduCDEGH* and *pduQ*	[43]
*C. beijerinkii* DSM 51	Source of *adh*	[44]
*K. pneumoniae* ATCC 9621	Reference strain	[15]
*K. pneumoniae* G31	NBIMCC 8645; overproducer of 2,3-BD	[22]
*K. pneumoniae* G31 K3	*K. pneumoniae* G31 with pCR_*pduC*^+^*DEGH*	This study
*K. pneumoniae* G31 K4	*K. pneumoniae* G31 with pCR_*pduC*^+^*DEGH_pduQ*^+^	This study
*K. pneumoniae* G31 K5	*K. pneumoniae* G31 with pCR_*pduC*^+^*DEGH*_T7_*pduQ*	This study
*K. pneumoniae* G31 K6	*K. pneumoniae* G31 with pCR_*pduC*^+^*DEGH*_T7_*adh*	This study
*K. pneumoniae* G31 K7	*K. pneumoniae* G31 with pCR_*pduC*^+^*DEGH*_P*tac*_*pduQ*	This study
*K. pneumoniae* G31 K8	*K. pneumoniae* G31 with pCR_*pduC*^+^*DEGH*_P*tac*_*adh*	This study
*K. pneumoniae* G31 K8_P*tac*_Xba	*K. pneumoniae* G31 with pCR_*pduC*^+^*DEGH*_P*tac*_XbaI_*adh*	This study
*K. pneumoniae* ATCC 9621 K6	*K. pneumoniae* G31 with pCR_*pduC*^+^*DEGH*_T7_*adh*	This study
*K. pneumonia* ATCC 9621 K8	*K. pneumoniae* G31 with pCR_*pduC*^+^*DEGH*_P*tac*_XbaI_*adh*	This study

**Table 4:** PCR polymerases and PCR conditions used in this study.

Component	Premix Taq	Q5
MgCl_2_	1.5 mM	2 mM
KCl	50 mM	50 mM
Primers	400 nM	500 nM
Initial denaturation	94 °C/1 min	98 °C/30 s
Hot Start	98 °C/10 s	98 °C/10 s
Elongation (temperature)	72 °C	72 °C
Elongation (speed)	1 kb/min	1 kb/30–40 s

**Table 5:** Real-Time qPCR primers used in this study.

Primer	Sequence (5′ -> 3′)	Product (bp)	Position in the Gene
16S_F	ACTGTGAGACAGGTGCTGC	100	110–128
16S_R	ACCGCTGGCAACAAAGGATA	100	209–190
rpoD_F	GACCCGTGAAGGCGAAATTG	100	333–352
rpoD_R	CAGGTAGGTGATCGCTTCCG	100	432–413
pduC_F	CGTGATAACACAATTGCCGGT	100	1018–1038
pduC_R	TTGGCACAATCCCACCGTTA	100	1117–1098
pduQ_F	ACCCACCGCTGCATAACAT	100	191–209
pduQ_R	CCGGTATCAATTGCCGAACC	100	290–271
adh_F	AATTGGCATTGGAGCTGTTGG	100	516–536
adh_R	CTCAACACAAATCGGCCTGC	100	615–596

**Table 6:** Heterologous genes used for the construction of the engineered 2-butanol biosynthesis pathway.

ID	Source	Size (bp)	Function	NCBI GenBank Accession No.
*pduCDEGH*	*L. diolivorans* DSM 14421	5274	Glycerol dehydratase operon + reactivase subunits	AZEY01000108
*pduQ*	*L. diolivorans* DSM 14421	1122	Propanol dehydrogenase	AZEY01000108
*adh*	*C. beijerinckii* DSM 51	1056	NADP-dependent alcohol dehydrogenase	PX999923

**Table 7:** PCR primers used in this study.

Primer	Sequence (5′-3′)	Product	Size (bp)
pCR-TOPO_R	TCTGAGGGCCCAATTCGCC	pCR^®^2.1-TOPO^®^ backbone	3830 ^1^
pCR-TOPO_F	GTACCAAGCTTGGCGTAATCATGG		
pduC+_F	*caggaaacagctatgaccatgattacgccaagcttggtac*TTGAAACGTCAAAAGAGATTTG	*pduCDEGH* operon + native promoter	5254 ^2^
pduC+_R	*aacctcccatgaacgtaatg*TTAACTCTTAAATGGCACTC		
pduQ+_F	*gagtgccatttaagagttaa*CATTACGTTCATGGGAGGTTTAATTTATG	*pduQ* gene + native promoter	1148
pduQ+_R	*tgtaatacgactcactatagggcgaattgggccctctaga*TTAACGGATTACTTTCTTGTAAATGTTG		
K4_pduQ_T7_R	GGAATTCGAATTTCTTCCAT*tctagaggtcctaattcgcccta*	K4 backbone	9212
K4_pduQ_T7_F	ACAAGAAAGTAATCCGTTAA*ttaactcttaaatggcactcatcttgctga*		
pduQ_T7_F	GGCGAATT**A**GG**A**CCTCTAGA*atggaagaaattcgaattccaaccaaagt*	*pduQ* gene	1162
pduQ_T7_R	GAGTGCCATTTAAGAGTTAA*ttaacggattactttcttgtaaatgttgcgc*		
K7_pduQ_Ptac_R	*tctagaggtcctaattcgcc*CTATAGT	K7 backbone	9252
K7_pduQ_Ptac_F	*ttcctcctattataactattacaaatcagatgtcaa*AAATT AAACCTCCCATGAACGTAATGTTA		
pduQ_Ptac_F	*ttgacatctgatttgtaatagttataataggaggaa*ATGGAAG AAATTCGAATTCCAACCAAAGT	*pduQ* + P*tac* promoter ^3^	1158 ^3^
pduQ_Ptac_R	*actatagggcgaattaggacctctaga*TTAACG GATTACTTTCTTGTAAATGTTGCGC		
K4_adh_R	*tctagaggtcctaattcgcc*CTATAGT	K4 backbone	9212
K4_adh_F	*ttaactcttaaatggcac*TCATCTTGCTG		
adh_T7_F	TGTAATACGACTCACTATAGGGCGAATT**A**GG**A**C CTCTAGA*atgaaaggttttgcaatgctaggtattaataagt*	*adh* gene	1056
adh_T7_R	*ctcgagtttttcagcaagatgagtgc*CATTTAAGAGT TAATTATAATATAACTACTGCTTT AATTAAGTCTTTTGGCTTGTCTTTC		
K8_adh_Ptac	*ttcctcctattataactattacaaatcagatgtcaa*AAATTAAACCTCCCATGAACGTAATGTTAACTC	K8 backbone	9252
adh_Ptac_F	*ttgacatctgatttgtaatagttataataggaggaa*ATGAAAGGT TTTGCAATGCTAGGTATTAATAAGT	*adh* gene + Ptac ^3^ promoter	1092 ^3^
adh_Ptac_R	*actatagggcgaattaggacctctaga*TTATAATATAACTA CTGCTTTAATTAAGTCTTTTGGCTTGTCTTTC		
K8adh_Ptac_XbaI_R	AAGCAGTAGTTATATTATAA TCTAGAGGTCCTAATTCGC	K8 backbone	9278
K8_F	CCTCCTATTATAACTATTACAAATCAGATGTCAA	K8 backbone	9278
adhPtac_XbaI_F	*ttgacatctgatttgtaatagttataataggaggtctaga*ATG AAAGGTTTTGCAATGCTAGG	*adh* + Ptac promoter + *Xba*I site	1096
adhPtac_XbaI_R	*ggcgaattaggacctctaga*TTATAATATAACTACTG CTTTAATTAAGTCTTTTGGCTTGTCTTTC		

^1^ 101 bp cut between *Kpn*I and *Xba*l sites; ^2^ *pduC*^+^ includes 51 bp upstream region (5′cctaaaaatgattttccatcatatattaagtaaccgggaggaggaacataa 3′); ^3^ P*tac* promoter in Gibson tail. Gibson tails are marked in lowercase and italics. Bases introduced to generate Shine–Dalgarno (RBS) sites are marked in bold; *Xba*I sites are underlined. Gibson tails are not included in amplicon size calculations.

## Data Availability

The nucleotide sequence of the *C. beijerinckii* DSM 51 *adh* gene is available in NCBI GenBank with accession no. PX999923.

## Supplementary Materials

The following supporting information can be downloaded at: https://www.mdpi.com/article/10.3390/ijms27062892/s1.
