# Supplementary material for: Direct Production of 2-Butanol from Glucose by Recombinant Klebsiella pneumoniae Strains
Source: Int J Mol Sci. 2026 Mar 23;27(6):2892. doi: 10.3390/ijms27062892 (PMC13026961; doi:10.3390/ijms27062892)
Supplement: Supplementary file 1 [file ijms-27-02892-s001.zip › ijms-4187020-supplementary.pdf]

Supplementary material

Table S1. Recombinant plasmid constructs used for engineering the 2-butanol biosynthetic pathway in *K. pneumoniae*.

Recombinant constructs	Description	Size (bp)
pCR®2.1-TOPO®	Cloning vector; Km^R, Amp^R; ColE1 *ori*; f1 *ori*; T7 promoter	3931
pCR_*pduCDEGH*⁺	*pduCDEGH* from *L. diolivorans* under the native *pduC* promoter	9212
pCR_*pduCDEGH*⁺_*pduQ*⁺	*pduCDEGH* and *pduQ* (*L. diolivorans*) with their native promoters	10,360
pCR_*pduCDEGH*⁺_*pduQ*_T7	*pduCDEGH* under *pduC* promoter; *pduQ* under T7 promoter	10,334
pCR_*pduCDEGH*⁺_*adh*_T7	*pduCDEGH* under *pduC* promoter; *adh* from *C. beijerinckii* under T7 promoter	10,268
pCR_*pduCDEGH*⁺_*pduQ*_P*tac*	*pduCDEGH* under *pduC* promoter; *pduQ* under P*tac* promoter	10,370
pCR_*pduCDEGH*⁺_*adh*_P*tac*	*pduCDEGH* under *pduC* promoter; *adh* from *C. beijerinckii* under the P*tac* promoter	10,304
pCR_*pduCDEGH*⁺_*adh*_P*tac*_X	Same as above, with an *Xba*I site introduced between the RBS and ATG of *adh*	10,308
